# Role of Backyard Flocks in Transmission Dynamics of Highly Pathogenic Avian Influenza A(H5N8) Clade 2.3.4.4, France, 2016–2017

**DOI:** 10.3201/eid2503.181040

**Published:** 2019-03

**Authors:** Marie Souvestre, Claire Guinat, Eric Niqueux, Luc Robertet, Guillaume Croville, Mathilde Paul, Audrey Schmitz, Anne Bronner, Nicolas Eterradossi, Jean-Luc Guérin

**Affiliations:** Ecole Nationale Vétérinaire, Institut National de la Recherche Agronomique, Université de Toulouse, Toulouse, France (M. Souvestre, C. Guinat, L. Robertet, G. Croville, M. Paul, J.-L. Guérin);; Agence Nationale de Sécurité Sanitaire, Ploufragan, France (E. Niqueux, A. Schmitz, N. Eterradossi);; Direction Générale de l’Alimentation, Paris, France (A. Bronner)

**Keywords:** highly pathogenic avian influenza, risk factors, France, backyard flocks, biosecurity, viruses, poultry, H5N8, HPAI, clade 2.3.4.4, influenza, zoonoses

## Abstract

Highly pathogenic avian influenza A(H5N8) clade 2.3.4.4 spread in France during 2016–2017. We assessed the biosecurity and avian influenza virus infection status of 70 backyard flocks near H5N8-infected commercial farms. One flock was seropositive for clade 2.3.4.4. Backyard flocks linked to commercial farms had elevated risk for H5 infection.

In the past 2 years, major outbreaks of highly pathogenic avian influenza (HPAI) occurred in Europe, resulting in severe socioeconomic effects on the poultry industry (*1*,*2*). During November 28, 2016–March 23, 2017, a total 484 HPAI poultry outbreaks associated with influenza A(H5N8) clade 2.3.4.4 viruses of Eurasia A/goose/Guangdong/1/1996 lineage were reported in France (*2*). Virus introduction into the index farm probably was associated with wild birds; however, other transmission pathways for virus spread between farms have been considered, including trade-related movements and spatial proximity (*2*). Although most outbreaks occurred in commercial flocks (n = 464), outbreaks in ≈20 backyard flocks also were reported (*2*). Backyard flocks are generally assumed to be at risk for avian influenza virus (AIV) introduction from wildlife and from nearby commercial poultry flocks during influenza outbreaks (*3**,**4*). Because little is known about the prevalence of AIV in backyard flocks contiguous to commercial farms, we aimed to quantify the seroprevalence of AIV and H5 subtype and to identify risk factors for infection in backyard flocks near commercial farms affected by HPAI H5N8 during the 2016–2017 epidemic.

## The Study

We conducted our study in Gers Department (1 of the 101 administrative units in France). Gers accounted for 19.8% (96/484) of the HPAI H5N8 outbreaks reported during the epidemic; 55.2% (53/96) of the Gers outbreaks were spatiotemporally clustered during December 11, 2016–January 4, 2017 (*2*). Our study targeted backyard flocks that were located within a 1-km radius from HPAI H5N8 outbreaks reported on commercial farms in Gers (n = 169) (Figure). At the time of our study, no backyard flock in Gers had been reported as HPAI infected.

**Figure:**
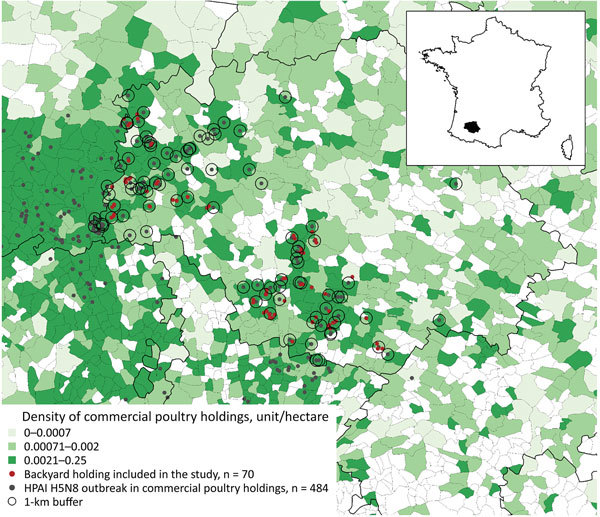
Locations of 484 commercial poultry holdings with reported outbreaks of HPAI H5N8 and the 70 backyard poultry holdings included in our study, Gers Department, France, 2016–2017. HPAI H5N8, highly pathogenic avian influenza A virus subtype H5N8.

Using a 28-question form, we conducted face-to-face interviews with each backyard flock owner during March 31–May 10, 2017. The 28 closed or semiclosed questions concerned the species of poultry, biosecurity practices, contacts with other flocks, and health status of the birds. We explained the purpose and methods of the study to all participants, who gave their consent to participate.

We sampled all backyard flocks up to a limit of 10 birds >6 months of age, which ensured that all sampled birds had been exposed to the HPAI outbreaks. Because flock size was as high as 60 birds (median 14 birds), detection thresholds ranged from 20% to 30% with a 95% CI. Not all flock owners consented to or were available for the study; in all, we were able to include 70 of the 169 backyard holdings.

We collected blood samples, tracheal swabs, and cloacal swabs. Blood was stored at 4°C after shipment, then serum was extracted and stored at –20°C. Tracheal and cloacal swabs were stored at –80°C until analysis. We performed serologic testing for AIV by using ELISA (IDVet ID Screen Influenza A Antibody Competition Multi-Species kit, http://www.id-vet.com). We considered a backyard flock as seropositive if >1 bird was found to be positive. We then tested AIV-seropositive backyard flocks for H5 antibodies by using the same IDVet ELISA kit, and we used hemagglutination inhibition tests to detect clade 2.3.4.4 H5 or other H5 Eurasian viruses (Appendix Table 1). Finally, we individually tested all birds from seropositive backyard flocks for AIV gene M and subtype H5 by using reverse transcription PCR (*5**,**6*). We performed descriptive statistics to assess how seroprevalences of AIV and H5 subtype were affected by flock owners’ practices (Appendix).

Estimated overall flock-level seroprevalence was 25.7% (95% CI 16.9%–37.0%) for AIV and 11.4% (95% CI 5.9%–21.0%) for H5 (Table 1). Estimated overall bird-level seroprevalence was 5.9% (95% CI 4.3%–8.1%) for AIV and 3.3% (95% CI 2.1%–5.0%) for H5. All birds tested were PCR-negative for gene M and H5.

**Table 1:** Results of serologic assays for 70 backyard flocks and 608 birds, by bird species comprising the flock, Gers Department, France, 2016–2017

Species comprising flock	Avian influenza virus				Influenza A virus subtype H5		
	Positive	Total	Seroprevalence, % (95% CI)		Positive	Total	Seroprevalence, % (95% CI)
All backyard holdings	18	70	25.7 (16.9–37.0)		8	70	11.4 (5.9–21.0)
Backyard holdings with only chickens	9	48	18.8 (10.2–31.9)		2	48	4.2 (1.2–14.0)
Backyard flocks with ducks	9	22	40.9 (23.3–61.3)		6	22	27.3 (13.2–48.2)
All birds	36	608	5.9 (4.3–8.1)		20	608	3.3 (2.1–5.0)
Chickens	20	486	4.1 (2.7–6.3)		9	486	1.9 (1.0–3.5)
Ducks	16	122	13.1 (8.2–20.2)		11	122	9.0 (5.1–15.4)

Among H5 ELISA-seropositive birds, only 3 belonging to the same flock showed positive hemagglutination inhibition titers against a clade 2.3.4.4 HPAI H5N8 antigen, and we could not confirm detection of clade 2.3.4.4–specific H5 antibodies with a second clade 2.3.4.4 H5N5 antigen in 1 of these birds. This backyard flock included chickens and ducks and was not adjacent to a commercial farm, and the owner reported working in a poultry meat processing plant.

Other H5 ELISA-positive birds were mainly seropositive for a couple of antigens from other H5 Eurasia lineages instead of clade 2.3.4.4 H5 HPAI virus. We could not distinguish between antibodies targeting low-pathogenicity or HPAI H5Nx viruses that spread in the region during 2015–2016 (*1*) (Appendix Table 1). This finding suggests that backyard flocks might have played a limited role in HPAI H5N8 transmission between farms during the 2016–2017 epidemic. Seroprevalence was higher in ducks than in chickens for AIV (13.1% [95% CI 8.2%–20.2%] vs. 4.1% [95% CI 2.7%–6.3%]) and H5 (9.0% [95% CI 5.1%–15.4%] vs. 1.9% [95% CI 1.0%–3.5%).

Backyard flocks that included ducks were more likely to be AIV-positive (odds ratio [OR] 2.3, 95% CI 1.1–5.1) and H5-positive (OR 5.7, 95% CI 1.6–30.6) than those having only chickens. These results are consistent with several studies emphasizing the role of ducks on AIV shedding and transmission (*1*). Specific attention was paid to flocks having ducks in the sampling design in the field because duck species could be considered as an additional risk factor (*1*). Thus, our study might overestimate the overall seroprevalence at the backyard flock and bird levels. Backyard flocks that had no fencing outdoors or had no covered food distribution area could be considered at higher risk for exposure to wild birds. However, these risk factors were not statistically associated with increased AIV or H5 seroprevalence (Appendix Table 2).

Backyard flocks located on or in close proximity to a commercial poultry farm were significantly more likely to be AIV-positive (OR 6.0, 95% CI 1.5–24.5) and H5-positive (OR 20.5, 95% CI 3.2–215.8). To date, proximity of commercial units to backyard flocks has not been considered as a risk factor, despite airborne transmission being suspected to spread disease (*7*,*8*). On the basis of the influenza A(H7N7) epidemic in the Netherlands, researchers constructed a model that assumed that infected backyard flocks were an example of spillover from commercial farms and that backyard flocks played no part in transmission (*9*). Our results highlight the importance of considering the impact of human activities in both the commercial and backyard flock settings. For commercial flocks, human activities have been described as a main source of secondary spread (*10*), with contacts through persons or shared equipment increasing the risk for AIV transmission (*11*). Consequently, a lack of biosecurity practices for backyard flocks belonging to commercial poultry farmers might have contributed to an increased risk for AIV infection of backyard poultry (Table 2).

**Table 2:** Variables included in the final multivariable logistic regression with avian influenza virus and influenza A virus subtype H5 seroprevalences as outcome variables, Gers Department, France, 2016–2017

Outcome and variable	Odds ratio (95% CI)	p value
Avian influenza virus		
Species included*	2.3 (1.1–5.1)	0.036
Link with poultry industry†	5.8 (1.5–24.5)	0.011
Influenza A virus subtype H5		
Species included*	5.7 (1.6–30.6)	0.019
Link with poultry industry†	20.5 (3.2–215.8)	0.003

*Backyard flocks having ducks (yes vs. no). †Professional activity of the backyard owner or member of the family home in connection with poultry industry (yes vs. no).

## Conclusions

We detected high flock- and bird-level seroprevalence of AIV in the backyard flocks we sampled after the 2016–2017 H5N8 epidemic in France. However, we observed very limited circulation of the H5N8 subtype, which indicates the minor role of backyard flocks in the transmission dynamics of H5N8. Backyard flocks belonging to commercial poultry farmers showed a significantly higher risk for infection with other H5 AIVs than backyard flocks having no links with commercial farms. These findings suggest that, from a risk-based perspective, surveillance of AIV circulation in backyard flocks should be focused on those flocks that have ducks and those connected to commercial poultry farms. On that basis, transmission of other more persistent pathogens of interest, such as mycoplasma or herpesviruses, should be further investigated at the backyard–commercial poultry interface (*12*).

Appendix Additional information on limited role of backyard flocks in the transmission dynamics of highly pathogenic avian influenza (H5N8) clade 2.3.4.4, France, 2016–2017.
