# Supplementary material for: Role of Backyard Flocks in Transmission Dynamics of Highly Pathogenic Avian Influenza A(H5N8) Clade 2.3.4.4, France, 2016–2017
Source: Emerg Infect Dis. 2019 Mar;25(3):551–4. doi: 10.3201/eid2503.181040 (PMC6390754; doi:10.3201/eid2503.181040)
Supplement: Appendix — Additional information on limited role of backyard flocks in the transmission dynamics of highly pathogenic avian influenza (H5N8) clade 2.3.4.4, France, 2016–2017. [file 18-1040-Techapp-s1.pdf]

Article DOI: https://doi.org/10.3201/eid2503.181040

# Role of Backyard Flocks in Transmission Dynamics of Highly Pathogenic Avian Influenza A(H5N8) Clade 2.3.4.4, France, 2016–2017

## Appendix

## Methods

Multivariable logistic regression analysis was conducted to investigate risk factors statistically associated with the infection status (AIV and H5). First, associations were tested between each outcome and explanatory variable using a chi-squared test or Fisher's test (Appendix Table 1). Explanatory variables with a p-value below 0.2 were selected to be included in multivariable logistic regressions. Pairwise collinearity was tested between all selected explanatory variables by computing Cohen's kappa coefficient and considered significant if the absolute value of the coefficient exceeded 0.7. For the multivariable regressions, stepwise backward elimination was performed and explanatory variables retained if statistically significant ($p<0.05$). All analyses were performed using R statistical software (version 3.4.1).

HIT (hemagglutination inhibition tests) were performed following international standards, using different couple of antigens specific for clade 2.3.4.4 H5 viruses, H5N8 A/decoyduck/France/161105a/2016 and H5N5 A/muteswan/ Croatia/102/2016, or for viruses belonging to other H5 Eurasian lineages, H5N3 A/muscovy duck/France/070090b/2007 and H5N2 A/chicken/France/03426a/2003 (http://www.oie.int/en/standard-setting/terrestrial-code/access-online). These pairs of H5 antigens, with different neuraminidase subtypes, were tested to exclude cross-reactivity driven by neuraminidase-specific antibodies.

## Antigens Used for HIT

H5N2 A/chicken/France/03426a/2003 and H5N3 A/muscovy duck/France/070090b/2007: sera displaying HI titers higher than 16 with both antigens are H5-positive in HIT. The antigens used have a broad cross-reactivity against H5 antibodies induced by infection or immunization with Eurasian lineage H5Nx low pathogenic or HPAI, except H5Nx A/goose/Guandong/1/1996-lineage clade 2.3.4.4 HPAI.

H5N5 A/muteswan/ Croatia/102/2016 (clade 2.3.4.4 A/goose/Guandong/1/1996 lineage) and H5N8 A/decoyduck/France/161105a/2016 (clade 2.3.4.4 A/goose/Guandong/1/1996 lineage): sera displaying HI titers higher than 16 with both antigens are H5-positive in HIT. The antigens used have a narrow cross-reactivity against H5 antibodies induced by infection or immunization with H5Nx A/goose/Guandong/1/1996-lineage clade 2.3.4.4 HPAI.

**Appendix Table 1.** ELISA, HIT and PCR analyses performed on AIV seropositive backyard poultry.

| Positive ELISA AIV backyards | Bird ID | Species | ELISA H5 | HIT H5N2 | HIT H5N3 | HIT H5N5 | HIT H5N8 | PCR M | PCR H5 |
|---|---|---|---|---|---|---|---|---|---|
| V5 | V5–02 | Poultry | – | ND | ND | ND | ND | – | – |
| V9 | V9–01 | Duck | + | <4 | <2 | 16 | 16 | – | – |
| | V9–02 | Poultry | + | ND | <2 | ND | 32 | – | – |
| | V9–03 | Duck | ± | <4 | <2 | 8 | 16 | – | – |
| | V9–09 | Duck | – | <4 | <2 | 8 | 8 | – | – |
| | V9–13 | Duck | – | <4 | <2 | <4 | <2 | – | – |
| V12 | V12–04 | Duck | – | ND | ND | ND | ND | – | – |
| V18 | V18–09 | Poultry | – | ND | ND | ND | ND | – | – |
| V21 | V21–04 | Duck | ± | <4 | <2 | <4 | <2 | – | – |
| | V21–06 | Poultry | + | ND | ND | ND | ND | – | – |
| V22 | V22–10 | Duck | ± | <4 | <2 | <4 | <2 | – | – |
| V24 | V24–04 | Poultry | – | ND | ND | ND | ND | – | – |
| V32 | V32–02 | Poultry | – | ND | ND | ND | ND | – | – |
| V33 | V33–02 | Poultry | – | ND | ND | ND | ND | – | – |
| | V33–08 | Poultry | – | ND | ND | ND | ND | – | – |
| V37 | V37–06 | Poultry | + | 16 | 32 | <4 | <2 | – | – |
| | V37–07 | Poultry | + | 16 | 16 | <4 | <2 | – | – |
| V50 | V50–03 | Poultry | + | <4 | 8 | <4 | <2 | – | – |
| V59 | V59–01 | Goose | + | 32 | 32 | <4 | <2 | – | – |
| | V59–02 | Goose | + | 16 | 16 | <4 | <2 | – | – |
| | V59–03 | Duck | + | 32 | 16 | <4 | <2 | – | – |
| | V59–05 | Duck | + | <4 | <2 | <4 | <2 | – | – |
| | V59–07 | Poultry | + | ND | <2 | ND | <2 | – | – |
| | V59–08 | Poultry | ± | <4 | <2 | <4 | <2 | – | – |
| | V59–09 | Poultry | + | <8 | 16 | <4 | <2 | – | – |
| | V59–10 | Poultry | + | 4 | 8 | ND | <2 | – | – |
| V62 | V62–03 | Goose | – | ND | ND | ND | ND | – | – |
| V69 | V69–01 | Duck | + | 32 | 8 | <4 | <2 | – | – |
| | V69–02 | Duck | + | <4 | <2 | <4 | <2 | – | – |
| V78 | V78–02 | Poultry | – | ND | ND | ND | ND | – | – |
| | V78–06 | Poultry | – | ND | ND | ND | ND | – | – |
| | V78–09 | Poultry | – | ND | ND | ND | ND | – | – |
| V81 | V81–08 | Duck | – | <4 | <2 | <4 | <2 | – | – |
| | V81–09 | Duck | + | <4 | <2 | <4 | <2 | – | – |
| V87 | V87–02 | Poultry | – | ND | ND | ND | ND | – | – |
| V85 | V85–03 | Poultry | – | ND | ND | ND | ND | – | – |

*HIT, hemagglutination inhibition test; ND, Not Done; +, positive results; –, negative results; ±, uncertain results.

**Appendix Table 2.** Binary variables examined for association with AIV and H5 seropositive holdings and results given by the univariate analysis with $p < 0.05$ * and $p < 0.01$ **.

| Variable | Description | Backyard holdings, % (proportion) | p value chi-squared or Fischer test AIV | p value chi-squared or Fischer test H5 |
|---|---|---|---|---|
| Species | Backyard flocks with ducks* | 31 (22/70) | 0.02* | 0.03* |
| Fenced outdoor | Access to a defined free-range area | 76 (53/70) | 0.99 | 0.99 |
| Covered backyard holding | Covered chicken house and/or free-range | 19 (13/70) | 0.19 | 0.19 |
| Covered food | Water and food distribution in a covered area | 57 (40/70) | 0.28 | 0.46 |
| Change practice | Modification of practices following the first H5 cases in South-Western France | 37 (26/70) | 0.38 | 0.70 |
| Clothes | No specific clothing for backyard care | 97 (68/70) | 0.99 | 0.99 |
| Shoes | No specific shoes for backyard care | 83 (58/70) | 0.43 | 0.71 |
| Handwashing | No handwashing before or after visiting the backyard | 33 (23/70) | 0.98 | 0.85 |
| Animal introduction | Bird introduction during the last year | 73 (51/70) | 0.92 | 0.70 |
| Backyard holdings' production | Selling or giving eggs to family or neighbors | 74 (52/70) | 0.51 | 0.51 |
| Backyard holdings' age | Owners having backyards holdings for more than 30 y | 59 (41/70) | 0.15 | 0.33 |
| Bird exhibition visit | Visit of a bird exhibition during the last three months | 4 (3/70) | 0.99 | 0.31 |
| Link with poultry industry | Professional activity of the backyard owner or member of the family home in connection with poultry industry ** | 17 (12/70) | 0.01** | 0.01** |
| Farmer's assistance | Owner giving assistance to a poultry farmer | 7 (5/70) | 0.99 | 0.99 |
| Hunting | Owner or member of family home being a hunter | 31 (22/70) | 0.93 | 0.42 |
| Backyard holding proximity | According to the owner, close distance to another backyard holding (1km or less) | 74 (52/70) | 0.99 | 0.99 |
| Mortality | Abnormal mortality during the three last months | 4 (3/70) | 0.99 | 0.99 |
| Ducks' clinical signs | Clinical signs on ducks during last three months | 0 (0/70) | – | – |
| Poultry clinical signs | Clinical signs on poultry during last three months | 6 (4/70) | 0.99 | 0.99 |
| Veterinary consultation | Veterinary visit or consultation during the three last months | 1 (1/70) | 0.26 | 0.11 |
